# Vapocoolant spray application during intra-articular knee injection: myth or reality?: A prospective randomized controlled trial

**DOI:** 10.1097/MD.0000000000041704

**Published:** 2025-02-21

**Authors:** Çağdaş Pamuk, Resul Musaoğlu, Ümit Gök, Ülker Moralar

**Affiliations:** a Department of Orthopedics and Traumatology, Silivri Anadolu Special Hospital, İstanbul, Turkey; b Department of Orthopedics and Traumatology, Kocaeli State Hospital, Kocaeli, Turkey.

**Keywords:** anxiety, hyaluronic acid, injections, intra-articular, knee, osteoarthritis, pain management

## Abstract

**Background:**

Intra-articular knee injections play a significant role in the treatment of knee osteoarthritis. This study aims to investigate the effects of vapocoolant spray application during intra-articular injections on pain and anxiety compared with placebo and injections performed without any agent.

**Methods:**

This prospective randomized controlled trial was conducted to investigate the effect of vapocoolant spray application on pain associated with intra-articular injection compared with placebo and no analgesic agent. Three groups, each consisting of 55 patients, were formed randomly. Vapocoolant spray was applied to 1 group, a placebo was applied to 1 group, and no analgesic was applied to the other group, and the results were evaluated.

**Results:**

After exclusions from a total of 197 patients, 165 were included in the study. The demographic characteristics of the groups were similar. While there was no significant difference in pain levels during needle insertion between group 1 and group 2, the Visual Analog Scale scores of group 1 and group 2 were significantly lower compared with group 3 (*P* < .001).

**Conclusion:**

This study revealed that vapocoolant spray application had no significant advantage over placebo in terms of pain scores during intra-knee injection.

## 1. Introduction

Intra-articular injections are a frequently used treatment method across many age groups and for various medical conditions.^[1]^ They are commonly used to treat knee osteoarthritis as well as other conditions such as cartilage damage and meniscus degeneration. The goal of these injections, which may include corticosteroids, hyaluronic acid (HA), or platelet-rich plasma, is to reduce pain symptoms and improve function.^[2–4]^

Previous studies have reported that one of the most significant disadvantages encountered during intra-articular injection procedures is pain and anxiety during the injection. For this reason, the application of certain topical agents has been explored to mitigate injection-related pain. Among these, vapocoolant sprays and local anesthetic creams are the most commonly used.^[5,6]^

Studies have shown that the application of vapocoolant sprays and topical anesthetic creams during intravenous cannulation and venous low molecular weight heparin administration reduces pain compared with placebo and increases patient comfort. Similarly, studies comparing vapocoolant sprays with intradermal lidocaine application during venous catheterization have reported that the spray application significantly reduces pain.^[7–10]^

In recent years, it has been demonstrated that vapocoolant spray application before more invasive procedures such as spinal injections, glenohumeral joint injections, ultrasound-guided breast needle biopsies, and digital nerve blocks can reduce needle-stick pain.^[11–14]^

Conversely, some studies have reported that, contrary to expectations, there is no significant difference in pain and anxiety scores 1 minute after vapocoolant spray application during hand injections. In addition, some studies have reported rare but potential side effects such as freezing, contact dermatitis, and inhalation intoxication. Furthermore, some authors have expressed concerns about potential skin sterility when using a spray solution before injection.^[5,13,15,16]^

To our knowledge, no studies have investigated the effects of vapocoolant spray application during intra-articular knee injections with a placebo-controlled design. Based on this, we hypothesized that, contrary to common belief, vapocoolant spray application may not have a significant effect compared with placebo during intra-articular knee injections.

The aim of this study is to investigate the effects of vapocoolant spray application during intra-articular knee injections on pain and anxiety compared with placebo and injections performed without any agent.

## 2. Materials and methods

Ethical approval was obtained from the Ethic Committee of the Medipol University Istanbul, Turkey (No. E-10840098-772.02-6199) for the prospective, randomized, controlled trial. Written informed consent was obtained from patients or their guardians before participation in this study.

Patients with knee osteoarthritis who underwent intra-articular HA injections were enrolled in the study between November 1, 2022, and December 30, 2022.

Inclusion criteria were as follows: Patients with Kellgren-Lawrence grade II–III–IV knee osteoarthritis on radiological imaging and pain during knee movement were included.

Exclusion criteria were as follows: Patients were excluded if they had previous knee injections, used topical or oral painkillers within 24 hours, had neurological disorders that could cause sensory loss at the injection site, had signs of infection at the injection site, had coagulation problems, had limited consciousness that prevented Visual Analog Scale (VAS) assessment, refused to provide written consent, or were participating in another clinical trial.

### 2.1. Patients

The sample size calculation indicated that 49 participants per group were required for 80% power and an alpha of 0.05. Accounting for a potential 10% exclusion rate, 55 knees per group were included in the study. Patients were enrolled until each group reached 55 participants, excluding those who were randomized but later excluded. Enrollment was considered complete when each group reached 55 patients.

### 2.2. Randomization and masking

Patients were randomized using the random.org website and divided into 3 groups. Group before the injection, vapocoolant spray was applied to group 1, isotonic saline spray (placebo) was applied to group 2, and no medication was given to group 3 before the injection. The vapocoolant spray and placebo spray cans were placed in an identical cover, and the contents were applied in a way that would not be understood. The cans were at room temperature and were applied from a distance of 15 cm for 5 seconds immediately before injection.

### 2.3. Clinical outcomes

Before the injection, patients were asked to rate their anxiety levels on a scale of 0 to 10 (0: no anxiety, 10: completely anxious). Anxiety levels were categorized as follows:

- Very high: anxiety score > 7
- High: anxiety score 6 or 7
- Medium: anxiety score 4 or 5
- Low: anxiety score < 4

A 100-mm VAS scale was used to assess pain levels during intra-articular knee injection. Patients were asked to mark their pain level on the 100-mm line, with the left side representing “no pain” and the right side representing “worst pain imaginable.”

#### 2.3.1. Satisfaction

Patients were asked to score their level of satisfaction with the procedure between 0 and 10 (0: completely dissatisfied, 10: maximum satisfaction). Satisfaction was grouped as follows:

- Very high: satisfaction score > 7
- High: satisfaction score 6 or 7
- Medium: satisfaction score 4 or 5
- Low: satisfaction score < 4

### 2.4. Patient intervention and evaluation process

All patients were informed about the study protocol and provided informed consent. Preintervention anxiety levels were assessed using a 100-mm VAS. Injections were administered under aseptic conditions using an anterolateral parapatellar approach with the patient in a seated position. A 4-mL prefilled syringe of HA preparation (lightly cross-linked sodium hyaluronate, Monovisc® [Anika Therapeutics, MA]) was used.

All injections were performed by the same physician. Before injection, the injection site was cleaned with 10% povidone-iodine solution. Group 1 received vapocoolant spray from a distance of 15 cm, group 2 received saline spray (placebo), and group 3 received no analgesic agent. Postinjection, patients were advised to restrict activity for 24 hours, rest, and apply cold packs. Immediately after the procedure, patients were asked to complete pain and satisfaction scores. A nurse explained how to complete the VAS score, and the scores were recorded. Patients were scheduled for a follow-up visit 1 week later to assess the injection site for potential complications. A 100-mm VAS and the Western Ontario and McMaster Universities Osteoarthritis (WOMAC)^[17]^ Index were used to assess patients’ functional outcomes at baseline and 1-week postinjection.

### 2.5. Statistical analysis

A power analysis was performed to determine adequate sample size. Based on 80% power, 5% false positive rate, and 0.15 effect size, a minimum sample size of 49 knees per group was calculated as necessary. Accordingly, the study was designed to include 55 knees per group to account for a potential dropout rate of approximately 10%.

Continuous data are expressed as mean and standard deviation, and categorical data are expressed as frequency (n) and percentages. The Kolmogorov–Smirnov test (with Lilliefors correction) was applied to each continuous variable to determine the presence/absence of normal distribution. Baseline demographic characteristics and the mean improvement from baseline for each of the clinical outcomes were assessed at each follow-up visit. The 2 groups were compared in terms of continuous variables using the Student *t* test (for continuous data that were normally distributed) or the Mann–Whitney *U* test (for continuous data that were nonnormally distributed). The Pearson chi-square test was used to evaluate the differences in the distributions of categorical variables between the 2 groups. All analyses were performed using the SPSS ver. 21 statistical package program (SPSS Inc., Chicago). *P* values of <.05 were accepted to show statistically significant results.

## 3. Results

**Figure 1.**
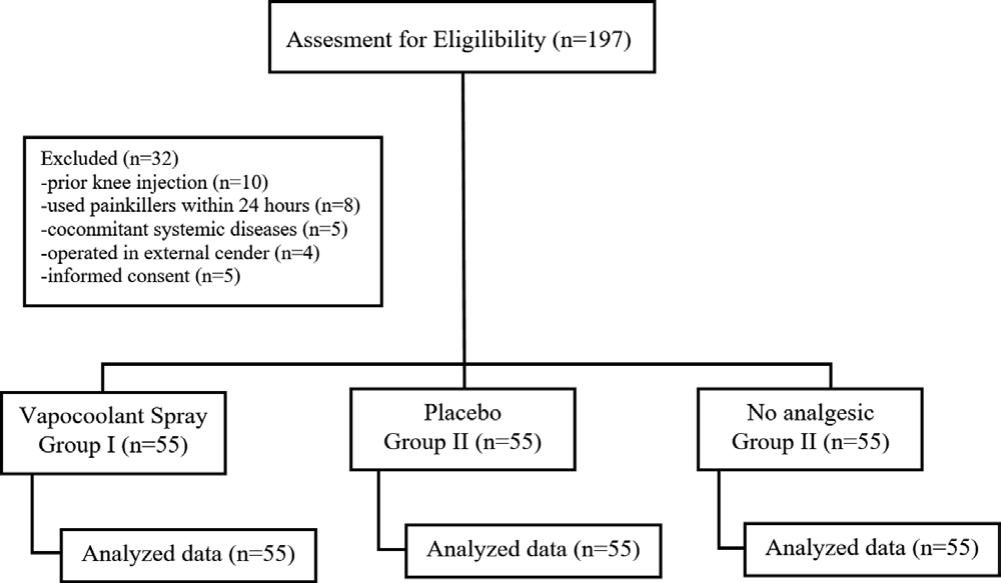
Consolidated Standards of Reporting Trial (CONSORT) diagram.

A total of 197 patients were evaluated and 32 were excluded from the study: 11 had a previous knee injection, 8 had used painkillers within 24 hours before the injection, 5 had systemic diseases, 4 were using systemic corticosteroids, and 5 refused to participate in the study (Fig. 1). Finally, 165 patients were included in the study, forming 3 groups of 55 patients each. Seventy-four patients (44.8%) were male, and the mean age was 55.8 ± 5.4 years. Patient demographics and baseline characteristics are shown in Table 1. There were no significant differences in demographic characteristics between the 3 groups.

**Table 1:** Demographic characteristics of patients.

	Group I (n = 55) Vapocoolan spray	Group II (n = 55) Placebo	Group III (n = 55) No analgesic	*P* value
Age	51.8 ± 9.4	57.9 ± 8.9	55.6 ± 9.6	.172
Sex (f:m)	13:42	19:36	17:38	.084
Body mass index	24.8 ± 3.2	25.4 ± 3.8	25.1 ± 3.4	.302
Kellgren-Lawrence grade				
2	39	33	37	.672
3	16	22	18	
Anxiety level				
* *Low	12(21.8%)	10(18.1%)	13(23.6%)	.344
* *Medium	24(43.6%)	26(47.2%)	21(38.1%)	
* *High	13(23.6%)	14(25.5%)	20(36.3%)	
* *Very high	6(10.9%)	5(9.1%)	1(1%)	
Knee pain (baseline)				
* *100-mm VAS score	62.5 ± 6.5	58.6 ± 7.2	60.8 ± 6.4	.482
Knee function (baseline)				
WOMAC total score	38.4 ± 10.6	37.2 ± 11.8	37.9 ± 10.8	.126

*P* value from Student *t* test, Pearson chi-square test, Mann–Whitney *U* test.

f:m = female:male, VAS = visual analog scale, WOMAC = Western Ontario and McMaster Universities Osteoarthritis Index.

There were no significant differences in anxiety levels between the groups. While there was no significant difference in needle-stick pain levels between group 1 and group 2, both groups had significantly lower VAS scores compared with group 3 (*P* < .001). Similarly, satisfaction levels showed no significant difference between group 1 and group 2, but both groups reported significantly higher satisfaction compared with group 3 (*P* < .001) (Table 2). No complications were observed during the 1-week follow-up. A significant improvement was observed in all groups after intra-articular HA injection when comparing baseline and 1-week functional outcomes (Table 3).

**Table 2:** Summary of clinical characteristics related to injection.

	Group I (n = 55) Vapocoolant spray	Group III (n = 55) No analgesic	*P* value	Group II (n = 55) Placebo	Group III (n = 55) No analgesic	*P* value	Group I (n = 55) Vapocoolant spray	Group II (n = 55) Placebo	*P* value
Anxiety									
Low	3 (5.45%)	4 (7.27%)	.384	6 (10.91%)	4 (7.27%)	.474	3 (5.45%)	6 (10.91%)	.296
Medium	8 (14.55%)	9 (16.36%)		7 (12.73%)	9 (16.36%)		8 (14.55%)	7 (12.73%)	
High	32 (58.18%)	29 (52.73%)		30 (54.55%)	29 (52.73%)		32 (58.18%)	30 (54.55%)	
Very high	12 (21.82%)	13 (23.64%)		12 (21.82%)	13 (23.64%)		12 (21.82%)	12 (21.82%)	
100-mm VAS score									
During needle penetration	42.6 ± 7.8	76.8 ± 7.3	**.018**	48.2 ± 6.2	76.8 ± 7.3	**.002**	42.6 ± 7.8	48.2 ± 6.2	.217
Satisfaction									
Low	4 (7.27%)	19 (34.55%)	**.036**	5 (9.09%)	19 (34.55%)	**.048**	4 (7.27%)	5 (9.09%)	.493
Medium	10 (18.18%)	24 (43.64%)		11 (20.00%)	24 (43.64%)		10 (18.18%)	11 (20.00%)	
High	30 (54.55%)	9 (16.36%)		29 (52.73%)	9 (16.36%)		30 (54.55%)	29 (52.73%)	
Very high	11 (20.00%)	3 (5.45%)		10 (18.18%)	3 (5.45%)		11 (20.00%)	10 (18.18%)	

Values are presented as mean ± standard deviation. Bold text indicates *P* < .05 and statistically significant findings.

VAS = visual analog scale, WOMAC = Western Ontario and McMaster Universities Osteoarthritis Index.

**Table 3:** Functional outcomes.

	Group I (n = 55) Vapocoolant Spray	Group II (n = 55) Placebo	Group III (n = 55) No analgesic	*P* value
100-mm VAS score				
Baseline	62.5 ± 6.5	58.6 ± 7.2	60.8 ± 6.4	.006
1 week	20.4 ± 5.5	23.8 ± 2.7	20.8 ± 4.7	
WOMAC total score				
Baseline	38.4 ± 10.6	37.2 ± 11.8	37.9 ± 10.8	.024
1 week	12.4 ± 10.1	14.3 ± 9.7	13.8 ± 6.8	

Values are presented as mean ± standard deviation. *P* value from Student *t* test, Mann–Whitney *U* test.

VAS = visual analog scale, WOMAC = Western Ontario and McMaster Universities Osteoarthritis Index.

## 4. Discussion

This study aimed to compare the use of vapocoolant spray to reduce pain during intra-articular HA injections for knee osteoarthritis with placebo and no analgesic treatment. Contrary to expectations, the vapocoolant spray showed no significant analgesic effect compared with the placebo. However, both the vapocoolant spray and placebo groups showed better pain scores and patient satisfaction compared with the injection group without any pretreatment. In addition, improvement in functional outcomes was observed within 1 week following intra-articular HA injection. As a general expectation, patients expect an application to reduce the pain caused by the needle. Therefore, we believe that the placebo effect meets the patient’s expectations and reduces their anxiety. The lack of a significant difference between placebo and vapocoolant spray suggests that placebo may simply be mechanically relieving pain and modulating patients’ anxiety levels.

Knee osteoarthritis is becoming increasingly common due to age-related degenerative processes. Conservative treatments are the first line of treatment, whereas intra-articular injections are often a second-line option. The effectiveness of intra-articular injections has been extensively studied, with various injection types compared in multiple studies.^[1–4]^ Although vapocoolant spray is frequently used clinically to reduce pain before intra-articular knee injections, its effectiveness has not been previously studied. The findings of our study suggest that vapocoolant spray does not provide a significant advantage over placebo in reducing pain.

Previous studies have investigated the use of vapocoolant spray for pain reduction during procedures such as subcutaneous injections and venous catheter placement, demonstrating improved pain and satisfaction levels.^[7,8,10,18]^ However, these studies often did not have both placebo and control groups. Our study, which included both placebo and control groups, shows that vapocoolant spray does not provide a significant analgesic effect compared with placebo.

Based on our current knowledge, this is the first study to compare vapocoolant spray with both placebo and a control group in the context of intra-articular injections. Although the use of vapocoolant spray for pain relief is not limited to injections, studies in the emergency department on early pain management of ankle trauma and rib fractures have shown its effectiveness in the posttraumatic setting. It has been shown to reduce pain and swelling, and to promote healing of the injured area.^[7,8,13,19]^ In trauma patients, cold application is a traditional method for managing bleeding and tissue edema associated with injury, and the efficacy of vapocoolant spray and cryotherapy is well established. However, we believe that injections differ from trauma cases because they often cause anxiety, which may affect pain perception.

Previous studies have examined variable pain perception in different body regions and have investigated the effects of vapocoolant spray before injections in the arm, leg, and abdomen, as well as the formation of hematomas and ecchymoses at the injection site. Intra-articular injections can be applied to almost any body region and are known to cause high anxiety in patients.^[9,15,18,20–22]^ This study focused only on the efficacy of vapocoolant spray during intra-articular knee injections. However, we predict similar results compared with placebo in other body regions.

In trauma patients, studies have shown that vapocoolant spray facilitates radiologic imaging during emergency department visits and enhances the analgesic effect of postoperative applications in extremity and abdominal surgery. However, both trauma and major postoperative cases differ from our study, which focused on anxiety-related pain during injections.^[6,23,24]^

Another important issue is the aseptic condition of the injection site. All intra-articular injections should be performed under absolutely sterile conditions with the utmost care. Prior spray application to the injection site may compromise sterility. However, previous studies investigating the effect of sprays on injection site sterility have found no evidence of contamination.^[25,26]^ Similarly, no deep or superficial infection was observed in our study.

Several studies have compared vapocoolant spray with topical anesthetic creams before injections, and many have shown that vapocoolant spray is ineffective or offers equivalent efficacy.^[7,10,12]^ Considering our findings, this suggests that vapocoolant spray may act as a placebo but does not have a direct significant effect.

Our study has several limitations. It included only patients with knee osteoarthritis who received intra-articular HA injections. Given the differences in pain perception in different body regions, we anticipate that intra-articular injections in other joints may yield different results. In addition, because the study focused on pain levels at the time of injection, long-term patient outcomes were not included. We did not include long-term injection results because we wanted to focus on the efficacy of the anesthetic application after the needle puncture and not the efficacy of the intra-articular injection.

In conclusion, vapocoolant spray showed no superiority over placebo in terms of pain scores during intra-articular HA injections in the knee. Patients undergoing intra-articular injections showed high anxiety levels. Both vapocoolant spray and placebo showed significantly better pain scores compared with injections without any pretreatment analgesia. This is the first study to evaluate the efficacy of vapocoolant spray compared with placebo in the context of intra-articular injections. Comparative studies with larger sample sizes and involving different joints are needed.

## Author contributions

**Conceptualization:** Çağdaş Pamuk, Resul Musaoğlu, Ülker Moralar.

**Data curation:** Çağdaş Pamuk, Ümit Gök, Ülker Moralar.

**Formal analysis:** Çağdaş Pamuk.

**Funding acquisition:** Çağdaş Pamuk, Resul Musaoğlu, Ülker Moralar.

**Investigation:** Çağdaş Pamuk, Ümit Gök.

**Methodology:** Çağdaş Pamuk, Resul Musaoğlu.

**Project administration:** Çağdaş Pamuk, Ümit Gök, Ülker Moralar.

**Resources:** Çağdaş Pamuk, Resul Musaoğlu.

**Software:** Çağdaş Pamuk, Ümit Gök.

**Supervision:** Çağdaş Pamuk, Resul Musaoğlu, Ülker Moralar.

**Validation:** Çağdaş Pamuk, Ümit Gök.

**Visualization:** Çağdaş Pamuk, Ümit Gök.

**Writing—original draft:** Çağdaş Pamuk, Ülker Moralar.
